# Acute Management of Substance Use Disorders: Clinical Outcomes and Predictors of Recovery in Emergency Psychiatric Care in Romania

**DOI:** 10.1192/j.eurpsy.2026.10843

**Published:** 2026-07-31

**Authors:** L. A. Moroianu, I. V. Dogaru, S.-D. Mitincu- Caramfil, M. Moroianu

**Affiliations:** 1 Medical Department; 2 Student; 3 Pharmaceutical Sciences Department; 4 Dental Medicine Deparment, University Dunarea de Jos, Galati, Romania

## Abstract

**Introduction:**

Substance use disorders represent a major public health challenge, with growing prevalence and complex interactions between biological, psychological, and social factors. Acute intoxication and withdrawal crises require rapid, multidisciplinary intervention to prevent severe complications and reduce mortality.

**Objectives:**

This study aimed to evaluate the effectiveness of pharmacological and non-pharmacological interventions in the acute management of patients with substance dependence admitted to the “Elisabeta Doamna” Psychiatric Hospital in Galați, Romania. Secondary aims included identifying demographic and clinical predictors of favorable outcomes and short-term relapse.

**Methods:**

A retrospective observational study was conducted between January 2020 and December 2024. Clinical records of **331 patients** hospitalized with a primary or secondary ICD-10 diagnosis of substance use disorder (F10–F19) were analyzed. Data included demographics, substance category, comorbidities, intervention type, and treatment response. Interventions were grouped as **pharmacological** (benzodiazepines, methadone/buprenorphine, antipsychotics, vitamin B complex, hepatoprotectors) or **combined** (pharmacological + psychological, social, and rehabilitative procedures). Statistical associations were tested using chi-square and descriptive analyses.

**Results:**

Of the 331 patients, **88.8 %** had a primary diagnosis and **11.2 %** a secondary diagnosis of substance dependence. The majority were **male (≈70 %)**, aged 36–50 years for alcohol dependence (F10.2) and 19–35 years for poly-drug cases (F19). Unemployment was the most frequent socioeconomic factor. Combined interventions were applied in **≈92 %** of cases, while pharmacologic therapy alone was applied in all. Laboratory or paraclinical assessments detected biological abnormalities in **≈39 %** of cases. The **clinical improvement rate** reached **98.3 %** in the combined-intervention group versus **76.3 %** for pharmacologic treatment alone (p < 0.01). Success was higher among younger patients, those without major comorbidities, and those engaged in psychotherapy or motivational counseling.

**Conclusions:**

Integrated management combining pharmacologic stabilization with psychological and social support significantly improves short-term recovery in patients with acute substance dependence. These findings highlight the importance of multidisciplinary collaboration and early psychosocial engagement in emergency settings. Results support the development of national clinical protocols and preventive policies targeting both medical and social determinants of addiction.

**Disclosure of Interest:**

None Declared

